# A model for identifying potentially inappropriate medication used in older people with dementia: a machine learning study

**DOI:** 10.1007/s11096-024-01730-0

**Published:** 2024-07-09

**Authors:** Qiaozhi Hu, Mengnan Zhao, Fei Teng, Gongchao Lin, Zhaohui Jin, Ting Xu

**Affiliations:** 1 Department of Pharmacy, West China Hospital, Sichuan University, Chengdu, China; 2 West China School of Medicine, Sichuan University, Chengdu, China; 3 School of Information Science and Technology, Southwest Jiaotong University, Chengdu, China

**Keywords:** Machine learning, Older dementia patients, Prescription, Potentially inappropriate medications

## Abstract

**Background:**

Older adults with dementia often face the risk of potentially inappropriate medication (PIM) use. The quality of PIM evaluation is hindered by researchers' unfamiliarity with evaluation criteria for inappropriate drug use. While traditional machine learning algorithms can enhance evaluation quality, they struggle with the multilabel nature of prescription data.

**Aim:**

This study aimed to combine six machine learning algorithms and three multilabel classification models to identify correlations in prescription information and develop an optimal model to identify PIMs in older adults with dementia.

**Method:**

This study was conducted from January 1, 2020, to December 31, 2020. We used cluster sampling to obtain prescription data from patients 65 years and older with dementia. We assessed PIMs using the 2019 Beers criteria, the most authoritative and widely recognized standard for PIM detection. Our modeling process used three problem transformation methods (binary relevance, label powerset, and classifier chain) and six classification algorithms.

**Results:**

We identified 18,338 older dementia patients and 36 PIMs types. The classifier chain + categorical boosting (CatBoost) model demonstrated superior performance, with the highest accuracy (97.93%), precision (95.39%), recall (94.07%), F1 score (95.69%), and subset accuracy values (97.41%), along with the lowest Hamming loss value (0.0011) and an acceptable duration of the operation (371s).

**Conclusion:**

This research introduces a pioneering CC + CatBoost warning model for PIMs in older dementia patients, utilizing machine-learning techniques. This model enables a quick and precise identification of PIMs, simplifying the manual evaluation process.

**Supplementary Information:**

The online version contains supplementary material available at 10.1007/s11096-024-01730-0.

## Impact statements

- Older patients with dementia frequently receive potentially inappropriate medications, leading to significant adverse drug events.
- Computer technology can enhance the quality of evaluation and the efficiency of identifying potentially inappropriate medications.
- Traditional machine learning algorithms struggle with the complexity of prescription data, making them less effective at identifying potentially inappropriate medications.
- A promising approach is to combine machine learning algorithms with multilabel classification models to uncover prescription data correlations and develop optimal models to identify potentially inappropriate medications in older adults with dementia.

## Introduction

The global demographic shift, marked by declining fertility rates and increasing life expectancy, has led to an aging population [1]. In the United States, projections suggest that by 2030, 1 in 5 Americans will be over 65 years old [2]. As of 2020, people aged 60 and older in China accounted for 18.7% of the population, totaling 264 million [3]. This population is particularly susceptible to neurodegenerative dementias, such as Alzheimer’s disease and dementia with Lewy bodies [3, 4]. Dementia, characterized by a severe decline in cognitive function hindering daily activities, is increasingly prevalent [4]. In 2020, an estimated 6.38 million Americans 65 years and older lived with Alzheimer's dementia, a figure expected to increase to 13.8 million by 2060 [3]. Similarly, a Chinese survey indicated that over 15.84 million people 60 years or older were affected by dementia in 2020 [4].

Comorbidity poses significant challenges in older patients, especially those with dementia, complicating disease management [5]. These patients are particularly vulnerable to the adverse effects of potentially inappropriate medications (PIMs). Common symptoms in dementia, such as agitation and sleep disturbances, often lead to the prescription of anticholinergics, antipsychotics, benzodiazepines, and Z drugs. Despite their initial perceived effectiveness, these medications carry risks such as cognitive decline, falls, hospitalization, and increased mortality, often overshadowing their benefits [6–8]. Evidence-based guidelines have been formulated to identify PIMs and medication indicators associated with higher adverse event risks in older adults [9]. PIMs, known for their unfavorable risk–benefit ratios, can exacerbate this population's risk of adverse events [10]. Prompt and effective identification of PIM is essential to enhance medical safety in older adults.

The Beers criteria, developed by the American Geriatrics Society (AGS), are widely used [11]. Its 2019 update classifies 99 PIMs into six groups [11]. Although these criteria have been applied in various studies to assess PIM occurrence in older patients across different diseases and regions, their effectiveness is often limited by researchers' familiarity with the criteria and the time-intensive nature of manual evaluations. Therefore, developing a computer algorithm for rapid and accurate identification of PIMs in older dementia patients is crucial.

Several clinical decision support systems (CDSSs) have been used to improve the appropriate prescribing for older patients [12–14]. However, these CDSSs identify PIMs based on keywords in the established database, which mean that these systems are less accurate for PIMs that contained unknown independent variables or are not targeted [12–14]. Therefore, a more efficient and intelligent approach should be applied to identify PIMs, especially for older patients with dementia.

Machine learning (ML) methods have increasingly become a tool of choice for medical researchers [15]. Numerous studies have developed predictive models based on singular data sources [16–18]. Extreme gradient boosting (XGBoost), categorical boosting (CatBoost), gradient-boosting decision tree (GBDT), light gradient-boosting machine (LightGBM) and random forest (RF) have been used to predict adverse drug events (ADEs) in older patient. These models achieved high accuracy in their predictions. However, prescription data often presents a multilabel problem, necessitating problem transformation methods. These methods improve model performance by converting the multilabel learning task into one or more single-label learning tasks [19].

**Fig. 1:**
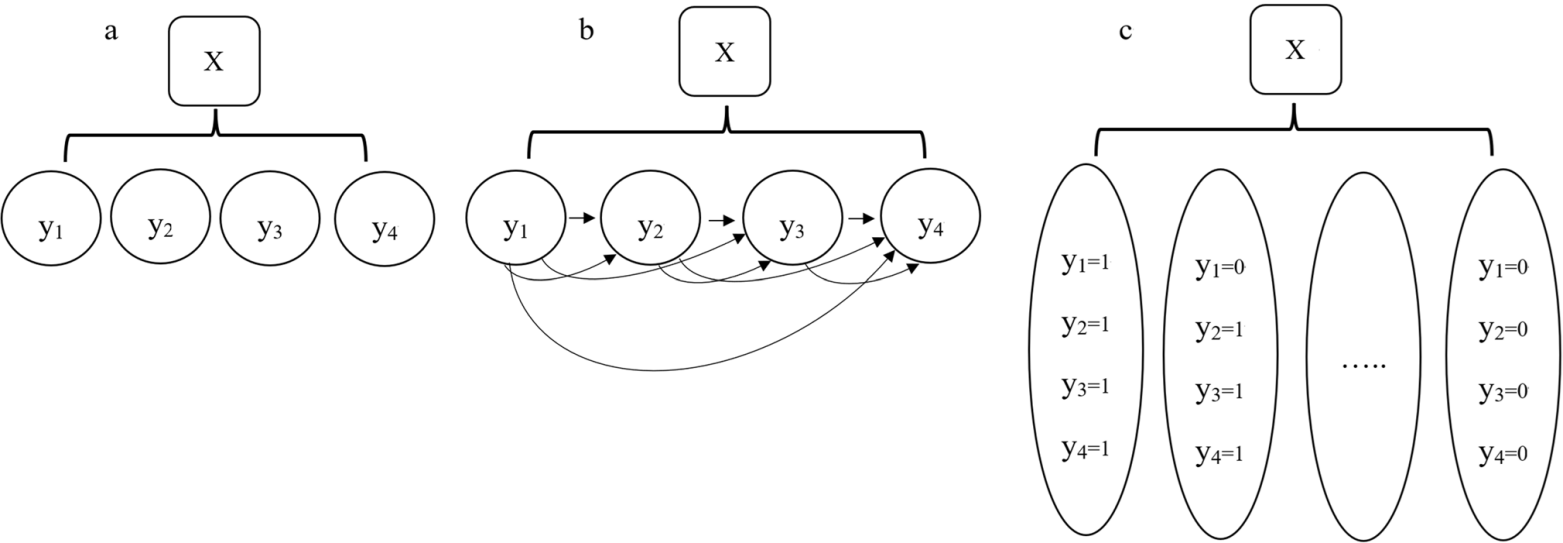
The process of multi-label classification problems. **a** Binary relevance; **b** Classifier chains; **c** Label power set, X: the data features, y_i_: the calculation results for the ith label

Multilabel classification (MLC) approaches are instrumental in identifying PIMs based on prescription information. Common MLC approaches include binary relevance (BR), label powerset (LP), and classifier chain (CC), as illustrated in Fig. 1. BR, a standard method, transforms the MLC task into several independent binary classification problems. However, it does not consider the label between the labels [20].

While BR efficiently optimizes Hamming Loss through one-step learning, it faces challenges with class imbalance, particularly in scenarios with low label cardinality and extensive label space [21]. In contrast, the LP method treats each combination of labels as a unique class, effectively converting the task into a multi-class problem. This approach, however, can lead to an exponential increase in the number of classes, making it time-consuming and potentially infeasible for large-label scenarios [22].

The CC approach enhances the BR method by implementing a high-order strategy to capture label interdependencies. It uses a chaining mechanism in which each classifier considers the predictions of its predecessors as additional inputs [23]. The process starts with a randomly chosen label to construct the first model. The predictions made using the CC approach on the entire dataset are then incorporated into the descriptor matrix. This matrix trains subsequent models on other randomly selected labels until all labels are addressed [22]. The effectiveness of the CC approach is mainly influenced by the random sequence of labels in the chain [23]. Given that each MLC approach has unique advantages and limitations, and its efficacy varies with the data type and ML techniques, assessing its performance with real-world data is crucial to understanding its applicability in different contexts.

### Aim

This study aimed to combine six ML algorithms and three MLC models to identify correlations in prescription information and develop an optimal model to identify PIMs in older Chinese adults with dementia.

### Ethics approval

The Ethics Committee of the West China Hospital of Sichuan University, China, approved this study, approval number 2020–651 (date of approval June 24, 2020).

## Method

### Study setting and the study population

Data were extracted from our previous research [24]. This study collected data from 75 hospitals in eight major Chinese cities: Chengdu, Beijing, Guangzhou, Shanghai, Shenyang, Tianjin, Zhengzhou, and Hangzhou. These cities represent five main geographical regions in China: East, West, North, South, and Central China [24].

The study included prescriptions for older adults (65 years and older) diagnosed with dementia from January 1, 2020, to December 31, 2020 [24]. Dementia diagnoses were identified using the International Classification of Diseases, 10th revision (ICD-10). This included codes for various types of dementia, including Alzheimer’s disease (F000-F002, F009, G300, G301, G308 and G309), vascular dementia (F010-F013, F018 and F019), dementia in Parkinson’s disease (F023), Huntington’s disease (F022), Pick’s disease or frontotemporal dementia (F020), HIV-related dementia (F024), Creutzfeldt–Jakob disease (F021) and unspecified dementias (F03X and F028). A cluster sampling method was used to extract prescription data from the Hospital Information System (HIS) [24].

### Data collection

Demographic and clinical data were collected from medical records. These data included sociodemographic information (such as region, hospital, department, patient sex, and age) and medical details (disease diagnosis, payment form, generic and trade name of medications, specification, dosage form, administration route, number of drugs, and dosage, and frequency of administration) [24]. However, certain prescription information, especially patient sex, therapeutic regimen (medications, dosage form, administration route, doses, and administration frequencies), and disease diagnosis, was often incomplete. This incompleteness posed a challenge in accurately identifying PIMs. Consequently, prescriptions without this crucial information were excluded to ensure the reliability of the findings [24].

### Data cleaning

Although diagnoses were made according to ICD-10 requirements, typographical errors or handwriting mistakes frequently resulted in the computer system incorrectly categorizing specific diagnoses. Therefore, the diagnoses were revised based on ICD-10 standards. The revision process involved merging identical diagnosis and adding appropriate punctuation marks to differentiate between similar diagnoses. For example, "severe dementia" and "dementia (severe)" represent the same condition but could be erroneously identified as distinct diseases by the computer. To mitigate this issue and enhance model performance, these similar diagnoses were consolidated under a single, accurately punctuated term, such as "dementia (severe)." Examples of these revisions are provided in Supplemental Table 1.

**Table 1:** Patient characteristics

Variable	Testing set (n = 3668)	Training set (n = 14,670)	Total (n = 18,338)	*P*
Mean age, SD (range)	80.96 ± 7.69 (65–103)	80.87 ± 7.68 (65–102)	80.90 ± 7.69 (65–103)	0.591
Mean number of diseases, SD (range)	2.58 ± 2.31 (1–24)	2.55 ± 2.32 (1–25)	2.56 ± 2.32 (1–25)	0.475
Mean number of medications, SD (range)	2.83 ± 2.22 (1–27)	2.83 ± 2.23 (1–30)	2.83 ± 2.27 (1–30)	0.998
Mean number of PIMs, SD (range)	1.30 ± 1.89 (0–10)	1.33 ± 1.90 (0–10)	1.31 ± 1.89 (0–10)	0.421
Sex				
Male	1613	6617	9230	0.221
Female	2055	8053	10,108	
Number of PIMs				
0	2201	8882	11,083	1.00
1	132	566	698	
2	467	1824	2291	
3	96	397	493	
4	490	1926	2416	
5	186	690	876	
6	40	168	208	
7	43	168	211	
8	11	28	39	
9	0	18	18	
10	2	3	5	

### Evaluation criteria

Two trained researchers independently evaluated the prescribed medications. Any discrepancies in their assessments were reconciled through consultation with a third researcher. The identification of PIMs was based on the AGS 2019 Beers criteria [11]. However, due to the absence of renal function data in prescription records, the criterion related to PIMs based on estimated glomerular filtration rate (the fifth category of PIMs, from No.68 to No.90) was not applicable in this study (Supplemental Table 2) [11].

**Table 2:** Model performance

Problem transformation method	Classification model	Accuracy	Precision	Recall	F1	ss Acc	hm	Operation time (s)
BR	RF	0.9351	0.8569	0.8039	0.8242	0.9239	0.0030	288
	LightGBM	0.9460	0.8595	0.8905	0.8734	0.9357	0.0024	20
	XGBoost	0.9722	0.9430	0.9168	0.9293	0.9637	0.0013	358
	CatBoost	0.9782	0.9485	0.9366	0.9423	0.9706	0.0011	618
	DF	0.9708	0.9268	0.9226	0.9247	0.9635	0.0014	1423
	TabNet	0.9201	0.8254	0.8586	0.8379	0.9062	0.0045	69,631
CC	RF	0.9376	0.9111	0.7971	0.8432	0.9288	0.0033	144
	LightGBM	0.9441	0.8582	0.8906	0.8729	0.9346	0.0024	33
	XGBoost	0.9741	0.9477	0.9231	0.9349	0.9662	0.0013	358
	CatBoost	0.9793	0.9539	0.9407	0.9469	0.9741	0.0011	371
	DF	0.9719	0.9314	0.9190	0.9249	0.9659	0.0014	728
	TabNet	0.9212	0.8292	0.8372	0.8305	0.9125	0.0040	71,459
LP	RF	0.8920	0.8544	0.6599	0.7213	0.8882	0.0060	787
	LightGBM	0.5543	0.5206	0.5212	0.4482	0.5281	0.0615	294
	XGBoost	0.9362	0.8689	0.7850	0.8179	0.9305	0.0034	996
	CatBoost	0.9468	0.8881	0.8200	0.8478	0.9406	0.0030	6252
	DF	0.9329	0.8418	0.7817	0.8062	0.9267	0.0037	7850
	TabNet	0.8972	0.7353	0.7496	0.7416	0.8828	0.0091	448,505

BR: Binary relevance; CC: Classifier chain; LP: Label powerset; RF: Random forest; LightGBM: Light gradient boosting machine; XGBoost: Extreme gradient boosting; DF: Deep forest; ss Acc: Subset accuracy; hm: Hamming loss

### Model development

Data were randomly divided into two sets: a training set used for model development, and a testing set, used to evaluate the models' performance, in a ratio of 8:2. To address the MLC challenge presented by the prescriptions, we applied three methods: LP, CC, and BR. Subsequently, various ML algorithms, including CatBoost, XGBoost, LightGBM, GBDT, RF and TabNet, were used to create predictive models to identify PIMs. All data analyses were conducted using Python software, version 3.8.

### Model evaluation metrics

To evaluate and compare the performance of the models, several metrics were used, including accuracy, precision, recall, F1 scores, subset accuracy (ss Acc), and Hamming loss (hm) [25, 26]. The F1 score is the harmonic mean of precision and recall. Subset accuracy measures the proportion of instances where the predicted label subset matches the ground-truth label subset. hm quantifies the fraction of incorrectly classified example-label pairs.

### Statistical analysis

Statistical analyses were conducted using SPSS software (version 25.0) to identify significant differences between the training and testing sets. Categorical variables are summarized using counts and percentages, and continuous variables are presented as means with standard deviations (SDs) or medians with ranges, as appropriate. To compare the groups, the nonparametric Mann–Whitney U test was used for continuous variables, while the chi-square (χ^2^) test was used for categorical variables.

## Results

### Study population

A total of 55,904 electronic prescriptions were extracted. Excluded were made for various reasons: 286 prescriptions had incomplete diagnosis data, 1303 lacked patient sex information, 1185 were only solvents, and 385 contained repeated drugs. After randomly selecting, a total of 18,338 patients with dementia were enroll.

The mean age was 80.90 ± 7.69 years (65–103). Of these, 55.12% (10,108/18,338) were women. The median number of disease diagnoses per patient was 2 (1–25). A total of 15.88% (2912/18,338) of the prescriptions were for patients diagnosed with five or more diseases. The median number of medications prescribed was 2 (1–30), with 15.80% (2897/18,338) of the patients receiving five or more medications. After data cleaning, the identical diagnoses were consolidated, reducing the disease count from 1842 to 948,740 medicines were identified. PIMs were found in 7255 (39.56%) of the patients. Patient were divided into training and testing sets in an 8:2 ratio, comprising 14,670 and 3668, respectively. No significant differences were observed in any variables between the training and testing sets (*P* > 0.05), as shown in Table 1.

### Potentially inappropriate medication

**Fig. 2:**
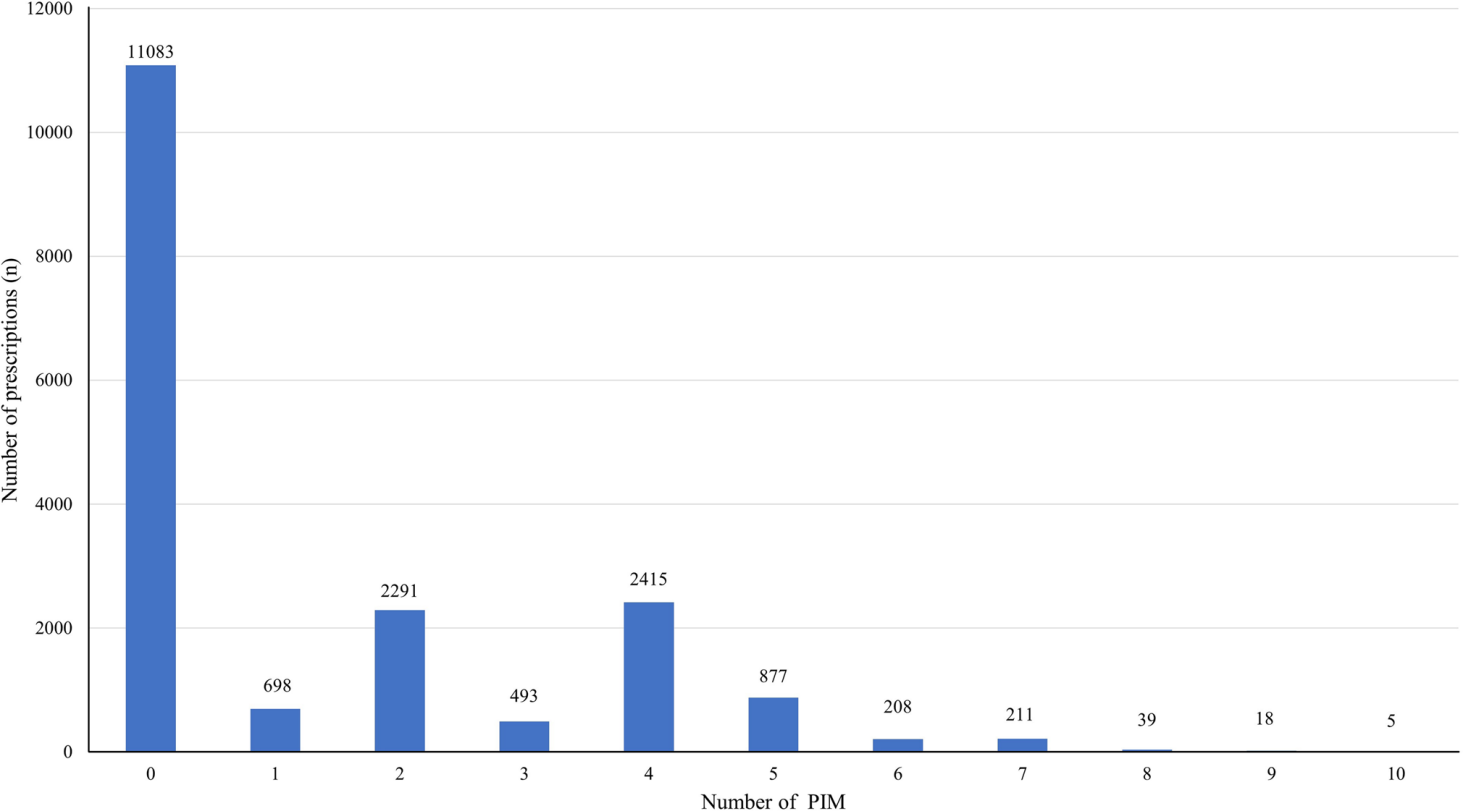
Frequencies of the number of PIMs prescribed

Among the 7225 patients prescribed PIMs, 24,053 PIMs were identified. Of these patients, 698 were patients one PIM, while 6557 received more than one PIM (Fig. 2). Thirty-six different types of PIMs were identified in these prescriptions [11].

**Fig. 3:**
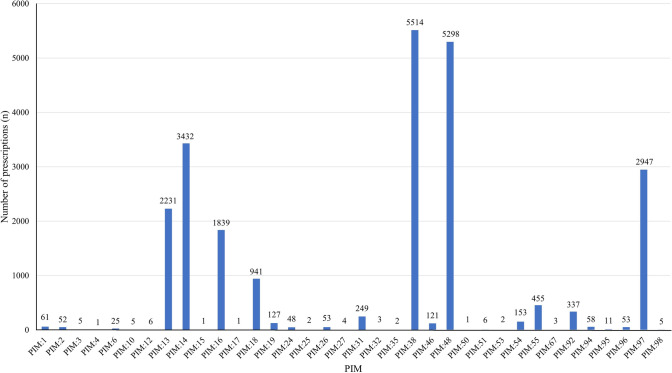
The frequencies of medications prescribed in each PIM

The most common PIMs included: No. 38 (avoid anticholinergics, benzodiazepines, benzodiazepine receptor agonist hypnotics, and antipsychotics in patients with dementia or cognitive impairment) (N = 5514; 22.92%); No. 48 (caution with antipsychotics, carbamazepine, diuretics, mirtazapine, oxcarbazepine, tramadol, or some types of antidepressants) (N = 5298; 22.03%); No. 14 (avoiding antipsychotics) (N = 3432; 14.27%); No. 97 (avoiding antipsychotics, including chlorpromazine, clozapine, loxapine, olanzapine, perphenazine, thioridazine, trifluoperazine) (N = 2947; 12.25%), and No. 13 (avoiding antidepressants) (N = 2231; 9.28%), as shown in Fig. 3.

Additionally, 11 types of PIMs, listed as Nos. 5, 7, 8, 9, 11, 28, 33, 47, 91, 93, and 99 (Supplemental Table 2), were not observed, likely because these drugs are either uncommonly used or not approved for use in China.

The comparative results of the six classification algorithms using MLC methods are presented in Table 2. The model using CC as the problem transformation method and CatBoost as the classification algorithm demonstrated superior outperformed over other models. The CC + CatBoost model achieved the highest accuracy (97.93%), precision (95.39%), recall (94.07%), F1 score (95.69%), and ss Acc values (97.41%), along with the lowest hm value (0.0011) and an acceptable operation duration (371s). Consequently, the CC + CatBoost model was chosen to identify PIMs. Table 3 presents the CC + CatBoost model's performance in evaluating each PIM within the test set.

**Table 3:** The performance of each PIM in the test set according to the CC + CatBoost model

No of PIM	Sample, n	Precision	Recall	F1
1	10	0.9995	1	0.8
2	15	1	1	1
3	2	0.9997	1	0.5
4	0	1	0	0
6	7	0.9995	1	0.7143
10	1	0.9997	0	0
12	1	1	1	1
13	460	0.9951	0.9804	0.9804
14	696	0.9954	0.9885	0.9871
15	0	1	0	0
16	372	0.9981	0.9867	0.9946
17	0	1	0	0
18	211	0.9986	0.9905	0.9858
19	23	1	1	1
24	12	1	1	1
25	0	1	0	0
26	11	1	1	1
27	2	1	1	1
31	46	0.9986	0.9767	0.913
32	0	1	0	0
35	0	1	0	0
38	1148	0.9959	0.9948	0.9922
46	26	0.9973	0.7667	0.8846
48	1090	0.9956	0.9954	0.9899
50	0	1	0	0
51	0	0.9997	0	0
53	0	1	0	0
54	29	0.9967	0.9048	0.6552
55	90	0.9945	0.9268	0.8444
67	0	1	0	0
92	68	0.9986	0.9701	0.9559
94	9	0.9997	1	0.8889
95	5	1	1	1
96	15	0.9997	0.9375	1
97	595	0.9984	1	0.9899
98	2	0.9997	1	0.5

## Discussion

Drug-related issues are a significant patient safety concern, especially prevalent in older adult with dementia [27]. Developing criteria to identify PIMs is essential to improve drug selection, educate clinicians and patients, and reduce ADEs. Globally recognized PIM criteria include the European Union (EU) (7) PIM list, Beers criteria and the STOPP/START criteria. The STOPP/START criteria comprise 80 STOPP and 34 START guidelines in their second version [28]. The EU (7) PIM list includes 282 substances or drug classes from 34 therapeutic groups [29]. The AGS Beers criteria, established in 2003 and updated every 3 to 4 years by the AGS Beers Criteria Expert Panel, are the most widely used standard for PIM detection [11].

Although Beers criteria are effectively used in clinical practice, they face challenges such as low efficiency and large heterogeneity [30]. CDSSs have been used to identify PIMs in older patients. However, studies indicate that while CDSSs are effective in hospitals, their performance varies in different ambulatory care settings [31]. These CDSSs, based on keyword-based identification from databases, applied in these studies were only able to identify part of the PIM, only detect a subset of PIMs, leading to potential non-detection issues [32, 33].

ML algorithms have gained widespread applications in medical fields, significantly enhancing the accuracy and efficiency of diagnosis, treatment [30], and prognosis prediction [34]. Compared to traditional modeling methods, ML offers distinct advantages in handling real-world evidence [35]. It can process complex, high-dimensional, and interactive variables more effectively, exhibiting stronger generalization capabilities and improved accuracy [35]. Consequently, ML algorithms are particularly adept at identifying PIMs in prescriptions with unknown independent variables. Advanced algorithms such as XGBoost, LightGBM, CatBoost, GBDT, and RF have been developed, offering refined techniques. XGBoost, LightGBM, CatBoost, GBDT belong to Gradient Boosting, a decision tree-based ensemble model. This iterative algorithm enhances its classifier by learning from the residual errors of previous trees, effectively reducing bias and variance in predictive models [36, 37]. In contrast, RF uses a bagging approach. It generates multiple bootstrap samples from the training data, with the final prediction being the average of all sub-model predictions [37].

We integrated three MLC approaches with six classification algorithms to develop a model to identify PIM prescription in older adults with dementia. Our results indicated that the CC + CatBoost model surpassed other models in performance. Introduced in 2017, CatBoost excels in handling categorical variables and incorporates automatic regularization to prevent overfitting [38]. The CC + CatBoost model demonstrated the highest accuracy, precision, recall, F1 score, and subset accuracy values, and the lowest hamming loss value. Therefore, it was chosen for PIM prescription identification in older dementia patients. With the advancement and proliferation of electronic medical record (EMR) system technology, ML models can increasingly integrate demographic and EMR data to identify PIM in older patients, particularly those with specific conditions such as dementia.

We found that certain PIMs were rarely infrequently, or even never, to older adults with dementia. In particular, 40 types of PIMs were absent from our study population. Among these, 12 types of PIMs were associated with potentially clinically significant drug-drug interactions. Clinicians in China typically avoid prescribing these combinations due to the risk of ADEs. For example, combinations such as warfarin with amiodarone or phenytoin with trimethoprim-sulfamethoxazole are generally avoided.

Other unobserved PIMs, such as the prescription of non-COX-2–selective nonsteroidal anti-inflammatory drugs to older patients with a history of gastric or duodenal ulcers, are typically avoided due to their association with ADEs. Consequently, these medications are seldom used in high-risk patient groups. Furthermore, 16 types of PIMs were rarely prescribed in this demographic attributed to the infrequent use of certain medications such as atropine (excluding ophthalmic use), belladonna alkaloids, and aclidinium-chlordiazepoxide.

Among the 20 commonly prescribed PIMs prescribed, antipsychotics were the most prescribed, especially in dementia patients. Numerous studies have cautioned against the use of antipsychotics in older patients with dementia or cognitive impairment due to the potential for cognitive function deterioration [39–41]. Consequently, managing the psychological symptoms of dementia should be highly individualized [39, 41]. Training caregivers has been recognized as the most effective intervention for these symptoms, with other nonpharmacological interventions also showing beneficial [38]. Antipsychotics should be reserved for cases where behaviors present a significant safety risk or when the patients with dementia experiences severe distress [39]. The serious adverse events associated with antipsychotic drugs, such as severe extrapyramidal effects and increased mortality, warrant cautious prescribing for dementia patients [40, 41]. However, the high prevalence of dementia in older Chinese patients, coupled with the shortage of caregivers and medical resources, may have contributed to antipsychotics becoming a standard first-line treatment for the psychological symptoms of dementia in this population.

Antidepressants and benzodiazepines were also frequently prescribed to dementia patients identified with PIMs. Among antidepressants, selective serotonin reuptake inhibitors (SSRIs), particularly citalopram and sertraline, were the most commonly used in this patient group. Studies have found not indicated significant differences between antidepressant and placebo for depressive symptoms in dementia patients [42], Furthermore, a higher risk of dementia has been associated with increased exposure to antidepressants [43]. Dementia is a progressive condition that affects memory, cognitive abilities and motor performance [44]. These impairments can inhibit daily activities and are associated with an increased risk of falls [44, 45]. Antidepressants, particularly SSRIs, can lead to hyponatremia and related adverse outcomes, such as impaired cognition, falls, fractures, and even mortality. The incidence of hyponatremia associated with SSRI use varies widely, ranging from 0.5 to 32% [46–50] and tends to increases with age [51]. The combined effect of the medication and the disease itself serves as a caution to physicians against prescribing antidepressants to older patients with dementia.

Several studies have suggested a link between benzodiazepines use and the onset of dementia [52–54]. Long-term benzodiazepines use is not only effectively in treating sleep disturbances but also increases the risks of cognitive impairment, delirium, falls, fractures, and motor vehicle crashes in older adults [55–57]. Although benzodiazepine receptor agonists and cognitive behavioral therapy are the recommended for chronic insomnia in older adults, their widespread application is currently challenging due to the large older population and limited medical resources.

Our study has both strengths and limitations. First, while the AGS Beers criteria are globally recognized, certain PIM types are rarely or never prescribed. The absence of these medications in our data set meant that the CC + CatBoost model’s performance could not be assessed for these rare PIM types. Future research aims to expand the knowledge domain and gather ample prescription data to enhance the model's performance. Second, access to personal and outpatient data, such as biochemical test results, patient height or weight, smoking history, medication history or the history of adverse drug reactions, may have led to underestimation or overestimation of the detection rate of PIMs. Third, several key aspects such as model validation, sample size validation, and Shapley Additive Explanations (SHAP) were not considered. Although the overall sample size was large, the sample size for PIMs was inadequate, potentially complicating the implementation of model validation. SHAP is valuable for elucidating the direction and significance of risk factors. However, due to the large number of risk factors involved, the application of SHAP could be challenging.

## Conclusion

This research introduces a CC + CatBoost warning model for PIMs in older dementia patients, utilizing ML techniques. This model enables a quick and precise identification of PIMs, simplifying the manual evaluation process.

### Supplementary Information

Below is the link to the electronic supplementary material.

Supplementary file1 (DOCX 16 KB)

Supplementary file2 (DOCX 42 KB)
