# Supplementary material for: A model for identifying potentially inappropriate medication used in older people with dementia: a machine learning study
Source: Int J Clin Pharm. 2024 Jul 9;46(4):937–46. doi: 10.1007/s11096-024-01730-0 (PMC11286713; doi:10.1007/s11096-024-01730-0)
Supplement: Supplementary file 1 — Supplementary file1 (DOCX 16 KB) [file 11096_2024_1730_MOESM1_ESM.docx]

Supplement Table 1. The example of data cleaning for diagnoses

| Before revision | After revision |
| --- | --- |
| Type 2 diabetes with complications | Type 2 diabetes with complications |
| Type 2 diabetes mellitus with multiple complications |  |
| Moderate to severe Alzheimer's disease | Dementia due to Alzheimer's disease (moderate to severe) |
| Moderate-to-severe Alzheimer's disease dementia |  |
| Moderate-to-severe Alzheimer's disease |  |
| Alzheimer's Disease dementia (severe) | Dementia due to Alzheimer's disease (severe) |
| Alzheimer's disease dementia is severe |  |
| Severe Alzheimer's dementia |  |
| Severe Alzheimer's disease dementia |  |
| Alzheimer's disease dementia is accompanied by psychobehavioral abnormalities | Alzheimer's disease dementia with psychobehavioral abnormalities |
| Alzheimer's dementia with hallucinatory delusional state |  |
| Alzheimer's dementia with other mental disorders |  |
| Alzheimer's disease dementia with psychobehavioral disorders |  |
| Severe dementia | Dementia (severe) |
| Dementia (severe) |  |
