# Supplementary material for: A model for identifying potentially inappropriate medication used in older people with dementia: a machine learning study
Source: Int J Clin Pharm. 2024 Jul 9;46(4):937–46. doi: 10.1007/s11096-024-01730-0 (PMC11286713; doi:10.1007/s11096-024-01730-0)
Supplement: Supplementary file 2 — Supplementary file2 (DOCX 42 KB) [file 11096_2024_1730_MOESM2_ESM.docx]

Supplement Table 2. The 2019 update of the AGS Beers Criteria

| ONE. Potentially inappropriate medication used in older adults | | | | |
| --- | --- | --- | --- | --- |
| No | Organ System, Therapeutic Category, Drug(s) | Rationale | Recommendation | |
| Anticholinergics | | | | |
| 1 | Anticholinergics： First-generation antihistamines Brompheniramine Carbinoxamine Chlorpheniramine Clemastine Cyproheptadine Dexbrompheniramine Dexchlorpheniramine Dimenhydrinate Diphenhydramine (oral) Doxylamine Hydroxyzine Meclizine Promethazine Pyrilamine Triprolidine | Highly anticholinergic; clearance reduced with advanced age, and tolerance develops when used as hypnotic; risk of confusion, dry mouth, constipation, and other anticholinergic effects or toxicity  Use of diphenhydramine in situations such as acute treatment of severe allergic reaction may be appropriate | Avoid | |
| 2 | Antiparkinsonian agents： Benztropine (oral) Trihexyphenidy | Not recommended for prevention or treatment of extrapyramidal symptoms with antipsychotics; more effective agents available for treatment of Parkinson disease | Avoid | |
| 3 | Antispasmodics： Atropine (excludes ophthalmic) Belladonna alkaloids Clidinium-chlordiazepoxide Dicyclomine Homatropine (excludes opthalmic) Hyoscyamine Methscopolamine Propantheline Scopolamine | Highly anticholinergic, uncertain effectiveness | Avoid | |
| Antithrombotics | | | | |
| 4 | Dipyridamole, oral short acting (does not apply to the extended-release combination with spirin) | May cause orthostatic hypotension; more effective alternatives available; IV form acceptable for use in cardiac stress testing | Avoid | |
| Anti-infective | | | | |
| 5 | Nitrofurantoin | Potential for pulmonary toxicity, hepatoxicity, and peripheral neuropathy, especially with long-term use; safer alternatives available | Avoid in individuals with creatinine clearance ＜ 30ml/min or for long-term suppression | |
| Cardiovascular | | | | |
| 6 | Peripheral alpha-1 blockers for treatment of hypertension Doxazosin Prazosin Terazosin | High risk of orthostatic hypotension and associated harms, especially in older adults; not recommended as routine treatment for hypertension; alternative agents have superior risk/benefit profile | Avoid use as an antihypertensive | |
| 7 | Central alpha-agonists： Clonidine for first-line treatment of hypertension Other CNS alpha-agonists Guanabenz Guanfacine Methyldopa Reserpine(＞0.1mg/day ) | High risk of adverse CNS effects; may cause bradycardia and orthostatic hypotension; not recommended as routine treatment for hypertension | Avoid other CNS alpha-agonists as listed | |
| 8 | Disopyramide | May induce heart failure in older adults because of potent negative inotropic action; strongly anticholinergic; other antiarrhythmic drugs preferred | Avoid | |
| 9 | Dronedarone | Worse outcomes have been reported in patients taking dronedarone who have permanent atrial fibrillation or severe or recently decompensated heart failure. | Avoid in individuals with permanent atrial fibrillation or severe or recently decompensated heart failure | |
| 10 | Digoxin for first-line treatment of atrial fibrillation or of heart failure | Use in atrial fibrillation: should not be used as a first-line agent in atrial fibrillation, because there are safer and more effective alternatives for rate control supported by high-quality evidence. Use in heart failure: evidence for benefits and harms of digoxin is conflicting and of lower quality; most but not all of the evidence concerns use in HFrEF. There is strong evidence for other agents as first-line therapy to reduce hospitalizations and mortality in adults with HFrEF. In heart failure, higher dosages are not associated with additional benefit and may increase risk of toxicity. Decreased renal clearance of digoxin may lead to increased risk of toxic effects; further dose reduction may be necessary in those with stage 4 or 5 chronic kidney disease. | Avoid this rate control agent as first line therapy for atrial fibrillation Avoid as first-line therapy for heart failure  If used for atrial fibrillation or heart failure, avoid dosages >0.125 mg/day Atrial fibrillation: low Heart failure: low Dosage ＞ 0.125 mg/d | |
| 11 | Nifedipine, immediate release | Potential for hypotension; risk of precipitating myocardial ischemia | Avoid | |
| 12 | Amiodarone | Effective for maintaining sinus rhythm but has greater toxicities than other antiarrhythmics used in atrial fibrillation; may be reasonable first-line therapy in patients with concomitant heart failure or substantial left ventricular hypertrophy if rhythm control is preferred over rate control | Avoid as first-line therapy for atrial fibrillation unless patient has heart failure or substantial left ventricular hypertrophy | |
| Central nervous system | | | | |
| 13 | Antidepressants, alone or in combination Amitriptyline Amoxapine Clomipramine Desipramine Doxepin >6 mg/day Imipramine Nortriptyline Paroxetine Protriptyline Trimipramine | Highly anticholinergic, sedating, and cause orthostatic hypotension; safety profile of low-dose doxepin (≤6 mg/day) comparable to that of placebo | Avoid | |
| 14 | Antipsychotics, first (conventional) and second (atypical) generation | Increased risk of cerebrovascular accident (stroke) and greater rate of cognitive decline and mortality in persons with dementia Avoid antipsychotics for behavioral problems of dementia or delirium unless nonpharmacological options (eg, behavioral interventions) have failed or are not possible and the older adult is threatening substantial harm to self or others | Avoid, except in schizophrenia or bipolar disorder, or for short-term use as antiemetic during chemotherapy | |
| 15 | Barbiturates Amobarbital Butabarbital Butalbital Mephobarbital Pentobarbital Phenobarbital Secobarbita | High rate of physical dependence, tolerance to sleep benefits, greater risk of overdose at low dosages | Avoid | |
| 16 | enzodiazepines Short and intermediate acting: Alprazolam Estazolam Lorazepam Oxazepam Temazepam Triazolam Long acting: Chlordiazepoxide (alone or in combination with amitriptyline or clidinium) Clonazepam Clorazepate Diazepam Flurazepam | Older adults have increased sensitivity to benzodiazepines and decreased metabolism of long acting agents; in general, all benzodiazepines increase risk of cognitive impairment, delirium, falls, fractures, and motor vehicle crashes in older adults May be appropriate for seizure disorders, rapid eye movement sleep behavior disorder, benzodiazepine withdrawal, ethanol withdrawal, severe generalized anxiety disorder, and periprocedural anesthesia | Avoid | |
| 17 | Meprobamate | High rate of physical dependence; sedating | Avoid | |
| 18 | Nonbenzodiazepine, benzodiazepine receptor agonist hypnotics (ie, “Z-drugs”) Eszopiclone Zaleplon Zolpidem | Nonbenzodiazepine benzodiazepine receptor agonist hypnotics (ie, Z drugs) have adverse events similar to those of benzodiazepines in older adults (eg, delirium, falls, fractures); increased emergency room visits/hospitalizations; motor vehicle crashes; minimal improvement in sleep latency and duration | Avoid | |
| 19 | Ergoloid mesylates (dehydrogenated ergot alkaloids) Isoxsuprine | Lack of efficac | Avoid | |
| Endocrine | | | | |
| 20 | Androgens Methyltestosterone Testosterone | Potential for cardiac problems; contraindicated in men with prostate cancer | Avoid unless indicated for confirmed hypogonadism with clinical symptoms Moderate Weak Desiccated thyroid | |
| 21 | Desiccated thyroid | Concerns about cardiac effects; safer alternatives available | Avoid | |
| 22 | Estrogens with or without progestins | Evidence of carcinogenic potential (breast and endometrium); lack of cardioprotective effect and cognitive protection in older women Evidence indicates that vaginal estrogens for the treatment of vaginal dryness are safe and effective; women with a history of breast cancer who do not respond to nonhormonal therapies are advised to discuss the risks and benefits of low-dose vaginal estrogen (dosages of estradiol <25 μg twice weekly) with their healthcare provider | Avoid systemic estrogen (eg, oral and topical patch) Vaginal cream or vaginal tablets: acceptable to use low-dose intravaginal estrogen for management of dyspareunia, recurrent lower urinary tract infections, and other vaginal symptoms | |
| 23 | Growth hormone | Impact on body composition is small and associated with edema,arthralgia, carpal tunnel syndrome, gynecomastia, impaired fasting glucose | Avoid, except for patients rigorously diagnosed by evidence-based criteria with growth hormone deficiency due to an established etiology | |
| 24 | nsulin, sliding scale (insulin regimens containing only short- or rapid-acting insulin dosed according to current blood glucose levels without concurrent use of basal or long-acting insulin) | Higher risk of hypoglycemia without improvement in hyperglycemia management regardless of care setting. Avoid insulin regimens that include only short- or rapid acting insulin dosed according to current blood glucose levels without concurrent use of basal or long-acting insulin. This recommendation does not apply to regimens that contain basal insulin or long-acting insulin | Avoid | |
| 25 | Megestrol | Minimal effect on weight; increases risk of thrombotic events and possibly death in older adults | Avoid | |
| 26 | Sulfonylureas, long acting Chlorpropamide Glimepiride Glyburide (also known as glibenclamide) | Chlorpropamide: prolonged half-life in older adults; can cause prolonged hypoglycemia; causes SIADH Glimepiride and glyburide: higher risk of severe prolonged hypoglycemia in older adults | Avoid | |
| Gastrointestinal | | | | |
| 27 | Metoclopramide | Can cause extrapyramidal effects, including tardive dyskinesia; risk may be greater in frail older adults and with prolonged exposure | Avoid, unless for gastroparesis with duration of use not to exceed 12 weeks except in rare cases | |
| 28 | Mineral oil, given orally | Potential for aspiration and adverse effects; safer alternatives available | Avoid | |
| 29 | Proton-pump inhibitors | Risk of Clostridium difficile infection and bone loss and fracture | Risk of Clostridium difficile infection and bone loss and fracture | |
| Pain medications | | | | |
| 30 | Meperidine | Oral analgesic not effective in dosages commonly used; may have higher risk of neurotoxicity, including delirium,than other opioids; safer alternatives available | Avoid | |
| 31 | Non–cyclooxygenase-selective NSAIDs, oral: Aspirin >325 mg/day Diclofenac Diflunisal Etodolac Fenoprofen Ibuprofen Ketoprofen Meclofenamate Mefenamic acid Meloxicam Nabumetone Naproxen Oxaprozin Piroxicam Sulindac Tolmetin | Increased risk of gastrointestinal bleeding or peptic ulcer disease in high-risk groups, including those >75 years or taking oral or parenteral corticosteroids, anticoagulants,or antiplatelet agents; use of proton-pump inhibitor or misoprostol reduces but does not eliminate risk. Upper gastrointestinal ulcers, gross bleeding, or perforation caused by NSAIDs occur in 1% of patients treated for 3-6 months and in 2%-4% of patients treated for 1 year; these trends continue with longer duration of use. Also can increase blood pressure and induce kidney injury.Risks are dose related. | Avoid chronic use, unless other alternatives are not effective and patient can take gastroprotective agent (proton-pump inhibitor or misoprostol) | |
| 32 | Indomethacin Ketorolac, includes parenteral | Increased risk of gastrointestinal bleeding/peptic ulcer disease and acute kidney injury in older adults Indomethacin is more likely than other NSAIDs to have adverse CNS effects. Of all the NSAIDs, indomethacin has the most adverse effects | Avoid | |
| 33 | Skeletal muscle relaxants Carisoprodol Chlorzoxazone Cyclobenzaprine Metaxalone Methocarbamol Orphenadrine | Most muscle relaxants poorly tolerated by older adults because some have anticholinergic adverse effects, sedation, increased risk of fractures; effectiveness at dosages tolerated by older adults questionable | Avoid | |
| Genitourinary | | | | |
| 34 | Desmopressin | High risk of hyponatremia; safer alternative treatments | Avoid for treatment of nocturia or nocturnal polyuria | |
| Two. potentially inappropriate medication used in older adults due to drug-disease or drug-syndrome interactions | | | | |
| No | Disease or Syndrome | Drug(s) | Rationale | Recommendation |
| Cardiovascula | | | | |
| 35 | Heart failure | Avoid: Cilostazol Avoid in heart failure with reduced ejection fraction: Nondihydropyridine CCBs (diltiazem,verapamil) Use with caution in patients with heart failure who are asymptomatic; avoid in patients with symptomatic heart failure: NSAIDs and COX-2 inhibitors Thiazolidinediones (pioglitazone, rosiglitazone) Dronedarone | Potential to promote fluid retention and/or exacerbate heart failure (NSAIDs and COX-2 inhibitors, nondihydropyridine CCBs, thiazolidinediones); potential to increase mortality in older adults with heart failure (cilostazol and dronedarone | As noted, avoid or use with caution |
| 36 | Syncope | AChEIs  Nonselective peripheral alpha-1 blockers (ie, doxazosin, prazosin, terazosin)  Tertiary TCAs Antipsychotics: Chlorpromazine Thioridazine Olanzapine | AChEIs cause bradycardia and should be avoided in older adults whose syncope may be due to bradycardia. Nonselective peripheral alpha-1 blockers cause orthostatic blood pressure changes and should be avoided in older adults whose syncope may be due to orthostatic hypotension. Tertiary TCAs and the antipsychotics listed increase the risk of orthostatic hypotension or bradycardia. | Avoid |
| Central nervous system | | | | |
| 37 | Delirium | Anticholinergics Antipsychoticsb Benzodiazepines Corticosteroids (oral and parenteral)c H2-receptor antagonists Cimetidine Famotidine Nizatidine Ranitidine Meperidine Nonbenzodiazepine, benzodiazepine receptor agonist hypnotics: eszopiclone, zaleplon, zolpidem | Avoid in older adults with or at high risk of delirium because of potential of inducing or worsening delirium Avoid antipsychotics for behavioral problems of dementia and/or delirium unless nonpharmacological options (eg, behavioral interventions) have failed or are not possible and the older adult is threatening substantial harm to self or others. Antipsychotics are associated with greater risk of cerebrovascular accident (stroke) and mortality in persons with dementia | Avoid |
| 38 | Dementia or cognitive impairment | Anticholinergics  Benzodiazepines Nonbenzodiazepine, benzodiazepine receptor agonist hypnotics Eszopiclone Zaleplon Zolpidem Antipsychotics, chronic and as-needed use | Avoid because of adverse CNS effects Avoid antipsychotics for behavioral problems of dementia and/or delirium unless nonpharmacological options (eg, behavioral interventions) have failed or are not possible and the older adult isthreatening substantial harm to self or others. Antipsychotics are associated with greater risk of cerebrovascular accident (stroke) and mortality in persons with dementia | Avoid |
| 39 | History of falls or fractures | Antiepileptics Antipsychoticsb Benzodiazepines Nonbenzodiazepine, benzodiazepine receptor agonist hypnotics Eszopiclone Zaleplon Zolpidem Antidepressants TCAs SSRIs SNRIs Opioids | May cause ataxia, impaired psychomotor function, syncope, additional falls; shorteracting benzodiazepines are not safer than long-acting ones. If one of the drugs must be used, consider reducing use of other CNS-active medications that increase risk of falls and fractures (ie, antiepileptics, opioid-receptor agonists, antipsychotics, antidepressants, nonbenzodiazepine and benzodiazepine receptor agonist hypnotics, other sedatives/hypnotics) and implement other strategies to reduce fall risk. Data for antidepressants are mixed but no compelling evidence that certain antidepressants confer less fall risk than others | Avoid unless safer alternatives are not available; avoid antiepileptics except for seizure and mood disorders  Opioids: avoid except for pain management in the setting of severe acute pain (eg, recent fractures or joint replacement) |
| 40 | Parkinson diseas | Antiemetics Metoclopramide Prochlorperazine Promethazine All antipsychotics (except quetiapine, clozapine, pimavanserin) | Dopamine-receptor antagonists with potential to worsen parkinsonian symptoms Exceptions: Pimavanserin and clozapine appear to be less likely to precipitate worsening of Parkinson disease. Quetiapine has only been studied in low-quality clinical trials with efficacy comparable to that and to that of clozapine in two others | Avoid |
| Gastrointestinal | | | | |
| 41 | History of gastric or duodenal ulcers | Aspirin >325 mg/day Non–COX-2–selective NSAID | May exacerbate existing ulcers or cause new/additional ulcers | Avoid unless other alternatives are not effective and patient can take gastroprotective agent (ie, proton-pump inhibitor or misoprostol) |
| Kidney/urinary tract | | | | |
| 42 | Chronic kidney disease stage 4 or higher (creatinine clearance <30 mL/min) | NSAIDs (non-COX and COX selective, oral and parenteral, nonacetylated salicylates) | May increase risk of acute kidney injury and further decline of renal function | Avoid |
| 43 | Urinary incontinence (all types) in wom | Estrogen oral and transdermal (excludes intravaginal estrogen) Peripheral alpha-1 blockers Doxazosin Prazosin Terazosin | Lack of efficacy (oral estrogen) and aggravation of incontinence (alpha-1 blockers) | Avoid in women |
| 44 | Lower urinary tract symptoms, benign prostatic hyperplasi | Strongly anticholinergic drugs, except antimuscarinics for urinary incontinence | May decrease urinary flow and cause urinary retention | Avoid in men |
| Three. drugs to be used with caution in older adults | | | | |
| No | Drug(s) | Rationale | Recommendation | |
| 45 | Aspirin for primary prevention of cardiovascular disease and colorectal cancer | Risk of major bleeding from aspirin increases markedly in older age. Several studies suggest lack of net benefit when used for primary prevention in older adult with cardiovascular risk factors, but evidence is not conclusive. Aspirin is generally indicated for secondary prevention in older adults with established cardiovascular disease. | Use with caution in adults ≥70 years | |
| 46 | Dabigatran Rivaroxaban | Increased risk of gastrointestinal bleeding compared with warfarin and reported rates with other direct oral anticoagulants when used for long-term treatment of VTE or atrial fibrillation in adults ≥75 years. | Use with caution for treatment of VTE or atrial fibrillation in adults ≥75 years | |
| 47 | Prasugrel | Increased risk of bleeding in older adults; benefit in highest-risk older adults (eg, those with prior myocardial infarction or diabetes mellitus) may offset risk when used for its approved indication of acute coronary syndrome to be managed with percutaneous coronary intervention. | Use with caution in adults ≥75 years | |
| 48 | Antipsychotics Carbamazepine Diuretics Mirtazapine Oxcarbazepine SNRIs SSRIs TCAs Tramadol | May exacerbate or cause SIADH or hyponatremia; monitor sodium level closely when starting or changing dosages in older adults | Use with caution | |
| 49 | Dextromethorphan/quinidine | Limited efficacy in patients with behavioral symptoms of dementia (does not apply to treatment of PBA). May increase risk of falls and concerns with clinically significant drug interactions. Does not apply to treatment of pseudobulbar affect. | Use with caution | |
| 50 | Trimethoprim-sulfamethoxazole | Increased risk of hyperkalemia when used concurrently with an ACEI or ARB in presence of decreased creatinine clearance | Use with caution in patients on ACEI or ARB and decreased creatinine clearance | |
| Four. potentially clinically important drug-drug interactions | | | | |
| No | Oject Drug and Class | Interacting Drug and Class | Risk Rationale | Recommendation |
| 51 | RAS inhibitor (ACEIs, ARBs, aliskiren) or potassium-sparing diuretics (amiloride, triamterene) | Another RAS inhibitor (ACEIs, ARBs, aliskiren) | Increased risk of hyperkalemia | Avoid routine use in those with chronic kidney disease stage 3a or high |
| 52 | Opioids | Benzodiazepines | Increased risk of overdose | Avoid |
| 53 | Opioids | Gabapentin, pregabalin | Increased risk of severe sedation-related adverse events, including respiratory depression and death | Avoid; exceptions are when transitioning from opioid therapy to gabapentin or pregabalin, or when using gabapentinoids to reduce opioid dose, although caution should be used in all circumstances |
| 54 | Anticholinergic | Anticholinergic | Increased risk of cognitive decline | Avoid; minimize number of anticholinergic drugs |
| 55 | Antidepressants (TCAs, SSRIs, and SNRIs) Antipsychotics Antiepileptics Benzodiazepines and nonbenzodiazepine, benzodiazepine receptor agonist hypnotics (ie, “Z-drugs”) Opioids | Any combination of three or more of these CNS-active drugs | Increased risk of falls (all) and of fracture (benzodiazepines and nonbenzodiazepine, benzodiazepine receptor agonist hypnotics) | Avoid total of three or more CNS-active drugsa; minimize number of CNS-active drug |
| 56 | Corticosteroids, oral or parenteral | NSAIDs | Increased risk of peptic ulcer disease or gastrointestinal bleeding | Avoid; if not possible, provide gastrointestinal protectio |
| 57 | Lithium | ACEIs | Increased risk of peptic ulcer disease or gastrointestinal bleeding | Avoid; if not possible, provide gastrointestinal protection |
| 58 | Lithium | Loop diuretics | Increased risk of lithium toxicity | Avoid; monitor lithium concentrations |
| 59 | Peripheral α-1 blockers | Loop diuretics | Increased risk of urinary incontinence in older women | Avoid in older women, unless conditions warrant both drugs |
| 60 | Phenytoin | Trimethoprim-sulfamethoxazole | Increased risk of phenytoin toxicity | Avoid |
| 61 | Theophylline | Cimetidine | Increased risk of theophylline toxicity | Avoid |
| 62 | Theophylline | Ciprofloxacin | Increased risk of theophylline toxicity | Avoid |
| 63 | Warfarin | Amiodarone | Increased risk of bleeding | Avoid when possible; if used together, monitor INR closel |
| 64 | Warfarin | Ciprofloxacin | Increased risk of bleeding | Avoid when possible; if used together, monitor INR closel |
| 65 | Warfarin | Macrolides (excluding azithromycin) | Increased risk of bleeding | Avoid when possible; if used together, monitor INR closel |
| 66 | Warfarin | Trimethoprim-sulfamethoxazole | Increased risk of bleeding | Avoid when possible; if used together, monitor INR closel |
| 67 | Warfarin | NSAIDs | Increased risk of bleeding | Avoid when possible; if used together, monitor INR closel |
|  |  |  |  |  |
| Five. medications that should be avoided or the dosage of which should be reduced with varying levels of kidney function in older adults (Not included in evaluation) | | | | |
| No | Medication Class and Medication | Creatinine Clearance at Which Action Required, mL/min | Rationale | Recommendation |
| Anti-infective | | | | |
| 68 | Ciprofloxacin | ＜ 30 | Increased risk of CNS effects (eg, seizures, confusion) and tendon rupture | Doses used to treat common infections typically require reduction when CrCl <30 mL/min |
| 69 | Trimethoprim-sulfamethoxazole | ＜ 30 | Increased risk of worsening of renal function and hyperkalemia | Reduce dose if CrCl 15-29 mL/min Avoid if CrCl <15 mL/min |
| Cardiovascular or hemostasis | | | | |
| 70 | Amiloride | ＜ 30 | Increased potassium and decreased sodium | Avoid |
| 71 | Apixaban | <25 | Lack of evidence for efficacyand safety in patients with a CrCl <25 mL/min | Avoid |
| 72 | Dabigatran | <30 | Lack of evidence for efficacy and safety in individuals with a CrCl <30 mL/min. Label dose for patients with a CrCl 15-30 mL/min based on pharmacokinetic data. | Avoid; dose adjustment advised when CrCl >30 mL/min in the presence of drug-drug interactions |
| 73 | Dofetilide | <60 | QTc prolongation and torsade de pointes | Reduce dose if CrCl 20-59 mL/min Avoid if CrCl <20 mL/min |
| 74 | Edoxaban | 15 ～ 50，＜ 15 or ＞ 95 | Lack of evidence of efficacy or safety in patients with a CrCl <30 mL/min | Reduce dose if CrCl 15-50 mL/min Avoid if CrCl <15 or >95 mL/min |
| 75 | Enoxaparin | <30 | Increased risk of bleeding | Reduce dose |
| 76 | Fondaparinux | <30 | Increased risk of bleeding | Avoid |
| 77 | Rivaroxaban | <50 | Lack of efficacy or safety evidence in patients with a CrCl <30 mL/min | Nonvalvular atrial fibrillation: reduce dose if CrCl 15-50 mL/min; avoid if CrCl <15 mL/min Venous thromboembolism treatment and for VTE prophylaxis with hip or knee replacement: avoid if CrCl <30 mL/min |
| 78 | Spironolactone | <30 | Increased potassium | Avoid |
| 79 | Triamterene | <30 | Increased potassium and decreased sodium | Avoid |
| Central nervous system and analgesics | | | | |
| 80 | Duloxetine | <30 | Increased gastrointestinal adverse effects (nausea,diarrhea) | Avoid |
| 81 | Gabapentin | <60 | CNS adverse effects | Reduce dose |
| 82 | Levetiracetam | ≤80 | CNS adverse effects | Reduce dose |
| 83 | Pregabalin | <60 | CNS adverse effects | Reduce dose |
| 84 | Tramadol | <30 | CNS adverse effects | Reduce dose; monitor for adverse effects |
| Gastrointestinal | | | | |
| 85 | Cimetidine | <50 | Mental status changes | Reduce dose |
| 86 | Famotidine | <50 | Mental status changes | Reduce dose |
| 87 | Nizatidine | <50 | Mental status changes | Reduce dose |
| 88 | Ranitidine | <50 | Mental status changes | Reduce dose |
| Hyperuricemia | | | | |
| 89 | Colchicin | <30 | Gastrointestinal, neuromuscular, bone marrow toxicity | Reduce dose; monitor for adverse effects |
| 90 | Probenecid | <30 | Loss of effectiveness | Avoid |
| Six. Drugs With Strong Anticholinergic Properties | | | | |
| No | Medication Class | Drug | Recommendation | |
| 91 | Antiarrhythmic | Disopyramide | Avoid | |
| 92 | Antidepressants | Amitriptyline, amoxapine, clomipramine, desicpramine, doxepine (> 6mg), imipramine, nortriptyline, paroxetine, protiline, tramipramine | Avoid | |
| 93 | Antiemetics | Perphenazine, promethazine | Avoid | |
| 94 | Antihistamines (first generation) | Bromopheniramine, carbisamine, chlorpheniramine, Cremartin, Cyproheptadine, dexbromopheniramine, dexchlorpheniramine, Zononin, Diphenhydramine (oral), antisensitivity, hydroxyzine, chlorine Phenylmethazine, Cli-librium, dicyclic amine, metatropine (excluding ophthalmic use), scopolamine, methylscopolamine, propylamine, promethazine, mepiramine, triplidine | Avoid | |
| 95 | Antimuscarinics(urinary incontinence) | Dafenaxin, Forstrodin, flavonoids piperidol, oxybutynin, Solinaxin, Tolterodine, trisloramine | Avoid | |
| 96 | Antiparkinsonian agents | Benzotropine, Trihexyphenidyl | Avoid | |
| 97 | Antipsychotics | Chlorpromazine, clozapine, loxapine, olanzapine, Perphenazine, thiridazine, triflurazine | Avoid | |
| 98 | Antispasmodics | Atropine (excl. ocular), Belladonna alkaloids, scopolamine (excl. ocular) | Avoid | |
| 99 | Skeletal muscle relaxants | Cyclobenzarine, Orphenadrine | Avoid | |
